# Supplementary material for: Revealing regional disparities in the transmission potential of SARS-CoV-2 from interventions in Southeast Asia
Source: Proc Biol Sci. 2020 Aug 26;287(1933):20201173. doi: 10.1098/rspb.2020.1173 (PMC7482285; doi:10.1098/rspb.2020.1173)
Supplement: Appendix 1 [file rspb20201173supp1.pdf]

Technical Appendix 1

Revealing regional disparities in the transmission potential of SARS-CoV-2 from interventions in South East A[illegible]

July 17, 2020

Contents

1 Change in Baseline Mobility In Residences 2

2 Residence-Only Model Fit Across Regions 2

3 Model Fit Across Regions 3

4 Residence-Only Kernel Coefficient for TVRN State Equation 3

5 Kernel Coefficients for TVRN State Equation 3

6 All Locality Regression-augmented TVRN 6

7 No Dorms Kernel Coefficients for TVRN State Equation 9

8 No Dorms Kernel TVRN State Equation 9

9 Sensitivity Analysis on Serial Interval 11
9.1 Serial Interval Estimates . . . 11
9.2 State Coefficient Estimates . . . 11
9.3 Model Fit . . . 11

10 Sensitivity Analysis on Incubation Period 12
10.1 Incubation Period Estimates . . . 12
10.2 State Coefficient Estimates . . . 12
10.3 Model Fit . . . 12

1 Change in Baseline Mobility In Residences

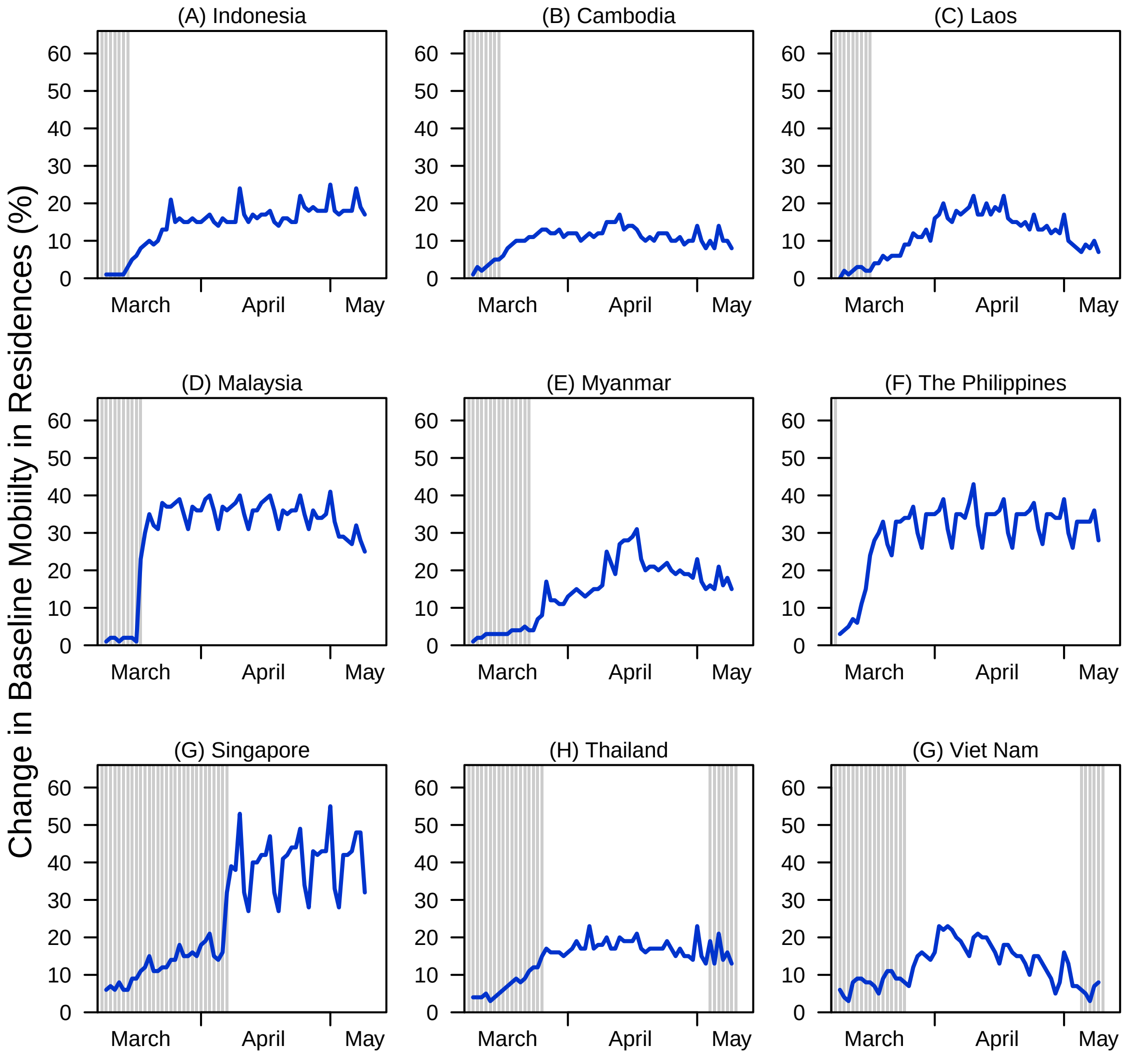

Change in baseline mobility in residences across 9 SEA countries. Time periods with no social distancing policy in place are indicated by gray regions.

2 Residence-Only Model Fit Across Regions

	MAE State	MAE Observation	R^2 State	R^2 Observation
Indonesia	0.20	49.92	0.86	0.85
Cambodia	0.13	2.16	0.86	0.22
Laos	0.04	0.33	0.95	0.12
Malaysia	0.30	44.94	0.85	0.50
Myanmar	0.12	2.48	0.74	0.21
Philippines	0.31	63.06	0.49	0.51
Singapore	0.28	136.05	0.15	0.76
Thailand	0.28	22.24	0.83	0.78
Vietnam	0.14	2.24	0.81	0.51

3 Model Fit Across Regions

	MAE State	MAE Observation	R^2 State	R^2 Observation
Indonesia	0.08	53.20	0.99	0.83
Cambodia	0.08	3.12	0.98	0.17
Laos	0.03	0.41	1.00	0.11
Malaysia	0.16	67.18	0.97	0.47
Myanmar	0.09	2.66	0.90	0.21
Philippines	0.25	64.46	0.91	0.50
Singapore	0.24	137.27	0.65	0.76
Thailand	0.18	34.03	0.97	0.79
Vietnam	0.09	2.59	0.97	0.52

Table 1: Model Fit Across Regions

4 Residence-Only Kernel Coefficient for TVRN State Equation

	Posterior Mean	Quantile 2.5%	Quantile 97.5%
Indonesia	-0.109	-0.127	-0.092
Cambodia	-0.154	-0.196	-0.110
Laos	-0.038	-0.072	-0.007
Malaysia	-0.074	-0.081	-0.066
Myanmar	0.034	0.017	0.053
Philippines	-0.041	-0.052	-0.031
Singapore	-0.009	-0.015	-0.004
Thailand	-0.145	-0.162	-0.128
Viet Nam	-0.065	-0.084	-0.046

5 Kernel Coefficients for TVRN State Equation

	Mean	Quantile 2.5%	Quantile 97.5%
Intercept	4.705	4.148	5.248
Retail Change From Baseline	0.054	-0.037	0.144
Grocery Change From Baseline	-0.052	-0.096	-0.009
Park Change From Baseline	-0.025	-0.080	0.030
Transit Change From Baseline	0.086	0.049	0.123
Workplace Change From Baseline	0.012	-0.024	0.048
Residential Change From Baseline	0.116	-0.024	0.256

Table 2: Indonesia

	Mean	Quantile 2.5%	Quantile 97.5%
Intercept	6.352	5.142	7.639
Retail Change From Baseline	-0.269	-0.365	-0.169
Grocery Change From Baseline	0.076	0.009	0.144
Park Change From Baseline	0.142	0.079	0.207
Transit Change From Baseline	0.198	0.135	0.263
Workplace Change From Baseline	0.061	0.032	0.090
Residential Change From Baseline	0.198	0.068	0.321

Table 3: Cambodia

	Mean	Quantile 2.5%	Quantile 97.5%
Intercept	2.078	0.929	3.085
Retail Change From Baseline	-0.129	-0.188	-0.043
Grocery Change From Baseline	0.010	-0.056	0.072
Park Change From Baseline	0.153	0.062	0.237
Transit Change From Baseline	0.083	0.038	0.119
Workplace Change From Baseline	0.059	0.027	0.089
Residential Change From Baseline	0.143	0.037	0.246

Table 4: Laos

	Mean	Quantile 2.5%	Quantile 97.5%
Intercept	5.734	5.281	6.184
Retail Change From Baseline	0.049	0.020	0.079
Grocery Change From Baseline	-0.047	-0.062	-0.033
Park Change From Baseline	-0.059	-0.091	-0.027
Transit Change From Baseline	0.111	0.075	0.147
Workplace Change From Baseline	-0.053	-0.076	-0.029
Residential Change From Baseline	-0.027	-0.081	0.029

Table 5: Malaysia

	Mean	Quantile 2.5%	Quantile 97.5%
Intercept	1.613	0.892	2.366
Retail Change From Baseline	-0.087	-0.144	-0.033
Grocery Change From Baseline	-0.103	-0.155	-0.056
Park Change From Baseline	-0.036	-0.092	0.019
Transit Change From Baseline	0.196	0.115	0.293
Workplace Change From Baseline	-0.053	-0.087	-0.020
Residential Change From Baseline	-0.105	-0.235	0.024

Table 6: Myanmar (Burma)

	Mean	Quantile 2.5%	Quantile 97.5%
Intercept	5.802	4.867	6.742
Retail Change From Baseline	0.008	-0.034	0.050
Grocery Change From Baseline	-0.011	-0.030	0.007
Park Change From Baseline	0.035	-0.003	0.073
Transit Change From Baseline	0.021	-0.013	0.055
Workplace Change From Baseline	-0.005	-0.041	0.032
Residential Change From Baseline	-0.038	-0.093	0.017

Table 7: Philippines

	Mean	Quantile 2.5%	Quantile 97.5%
Intercept	2.270	1.863	2.661
Retail Change From Baseline	-0.011	-0.045	0.024
Grocery Change From Baseline	-0.037	-0.053	-0.021
Park Change From Baseline	0.024	0.011	0.038
Transit Change From Baseline	0.019	-0.008	0.045
Workplace Change From Baseline	0.007	-0.014	0.030
Residential Change From Baseline	0.022	-0.009	0.054

Table 8: Singapore

	Mean	Quantile 2.5%	Quantile 97.5%
Intercept	6.272	5.740	6.814
Retail Change From Baseline	0.032	-0.003	0.069
Grocery Change From Baseline	-0.101	-0.126	-0.077
Park Change From Baseline	-0.079	-0.132	-0.025
Transit Change From Baseline	0.172	0.110	0.233
Workplace Change From Baseline	-0.063	-0.102	-0.024
Residential Change From Baseline	-0.060	-0.158	0.037

Table 9: Thailand

	Mean	Quantile 2.5%	Quantile 97.5%
Intercept	3.406	2.786	4.033
Retail Change From Baseline	-0.057	-0.088	-0.026
Grocery Change From Baseline	-0.109	-0.149	-0.067
Park Change From Baseline	0.034	0.004	0.063
Transit Change From Baseline	0.158	0.114	0.203
Workplace Change From Baseline	0.005	-0.010	0.018
Residential Change From Baseline	0.049	-0.015	0.114

Table 10: Vietnam

6 All Locality Regression-augmented TVRN

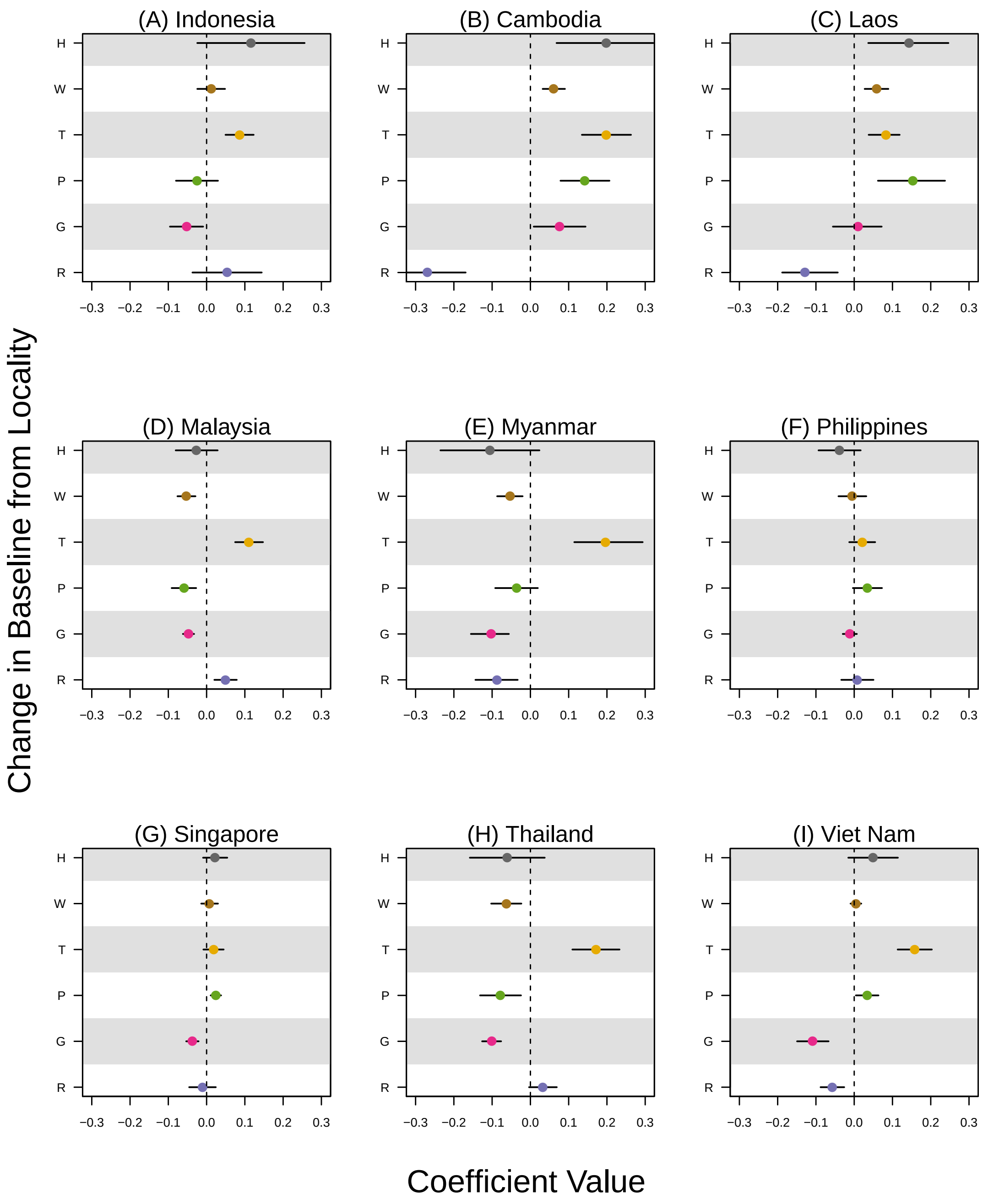

Figure 1: Regression-augmented TVRN state equation coefficients across 9 SEA countries with all CBM variables

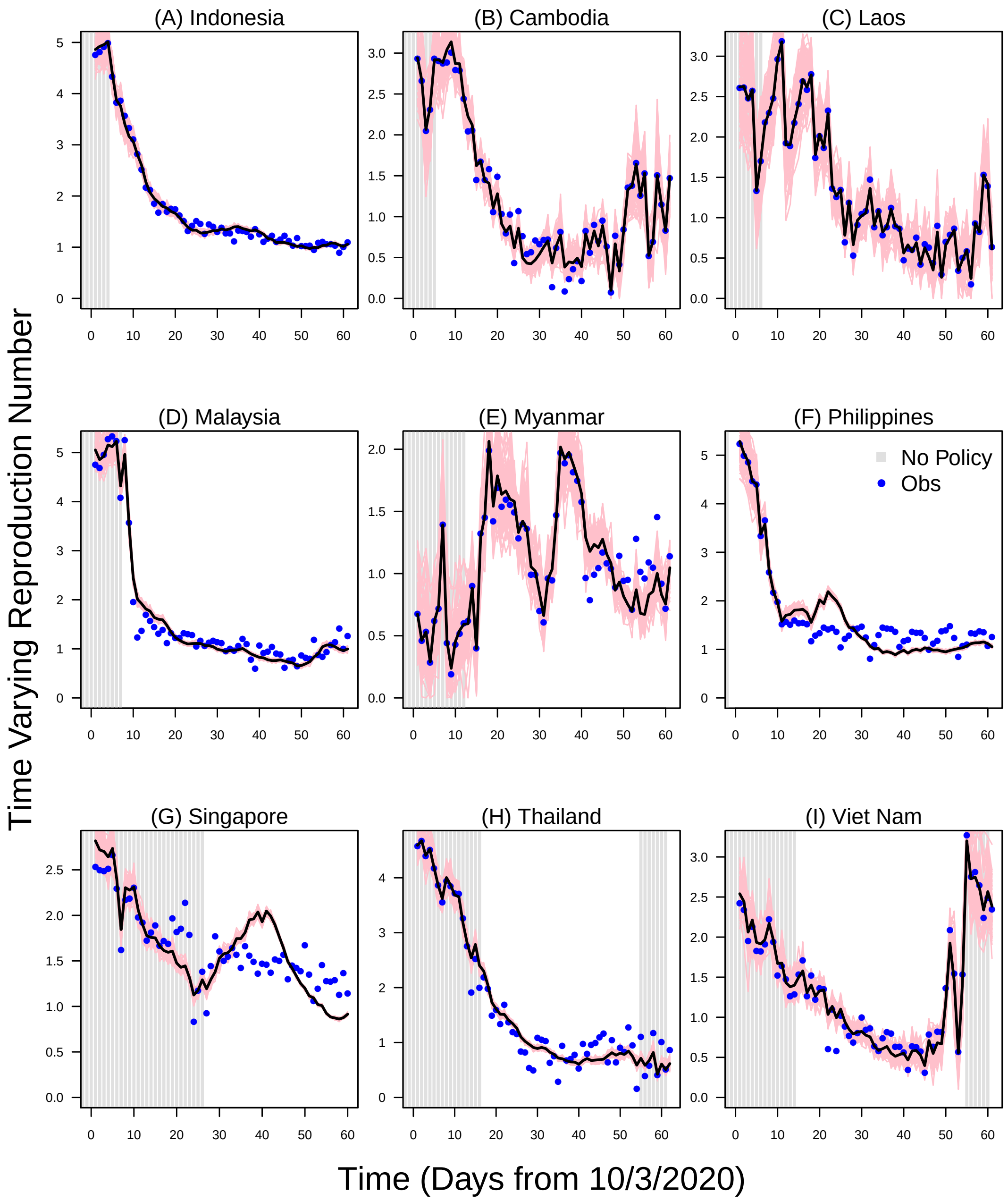

Figure 2: Regression-augmented TVRN across 9 SEA countries with all CBM variables

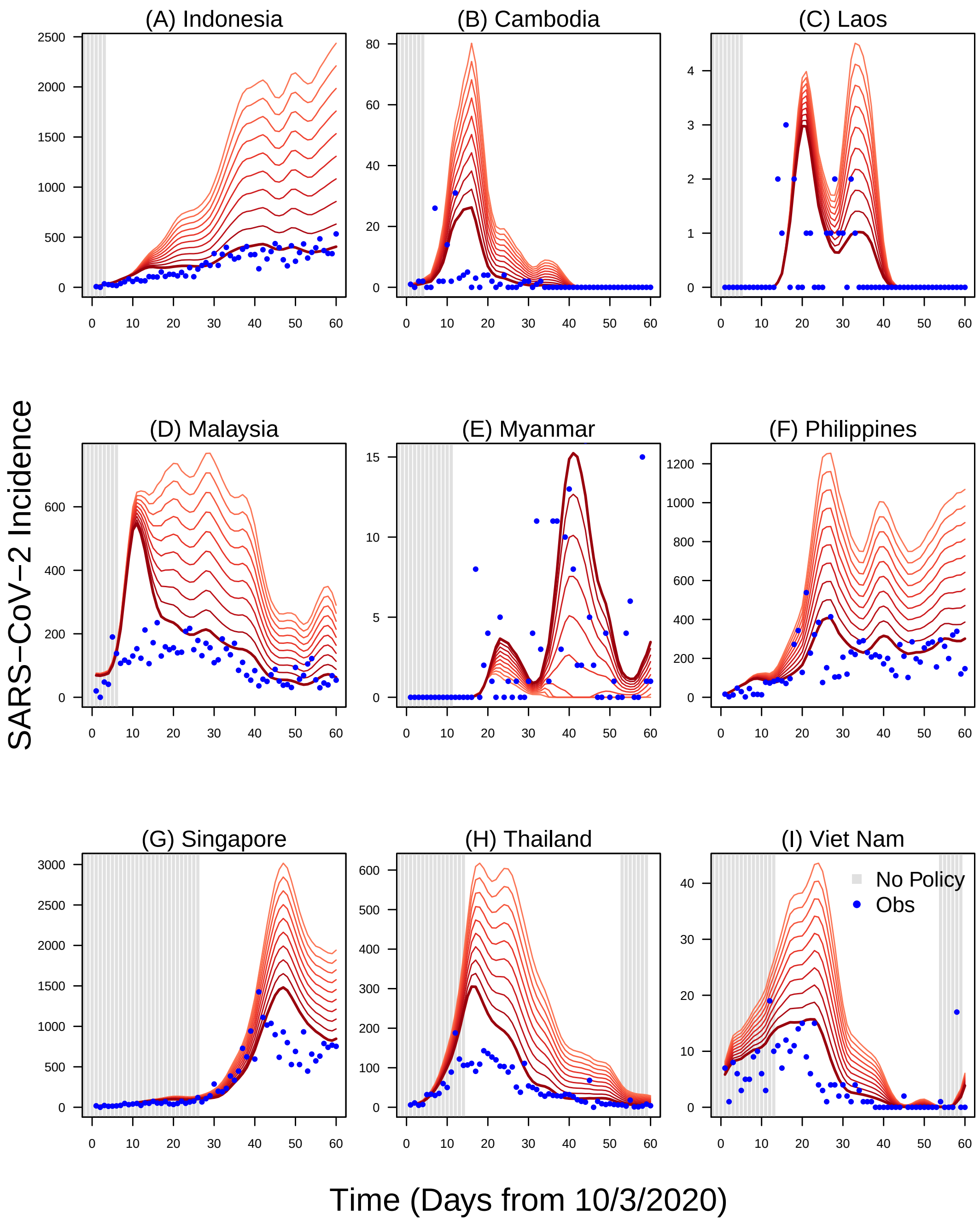

Figure 3: Regression-augmented TVRN interventions across 9 SEA countries

7 No Dorms Kernel Coefficients for TVRN State Equation

Singapore	Intercept	Residential Change From Baseline
Mean	0.80	-0.01
2.5 Quantile%	0.62	-0.02
97.5 Quantile%	0.98	-0.01

8 No Dorms Kernel TVRN State Equation

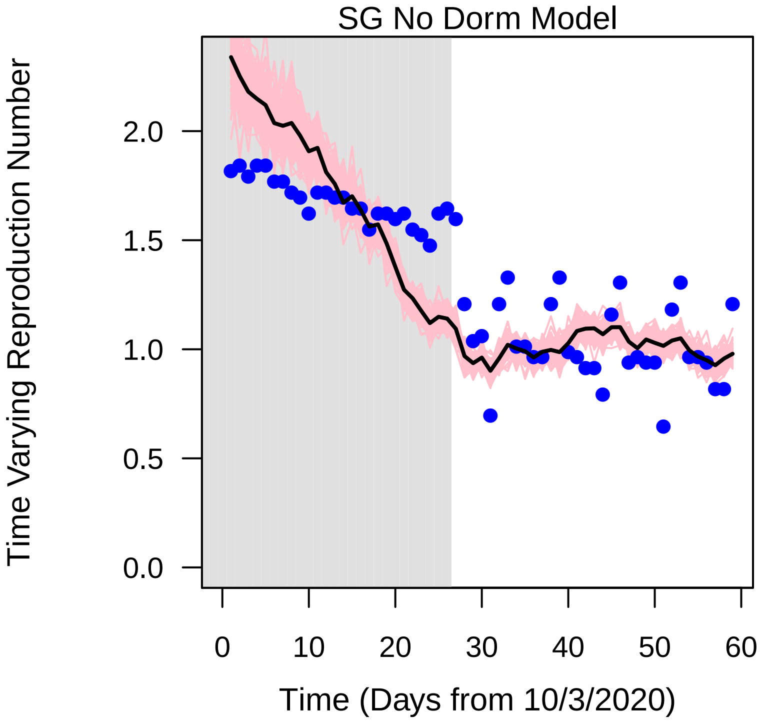

Regression-augmented TVRN for Singapore without COVID-19 Dormitory Case counts

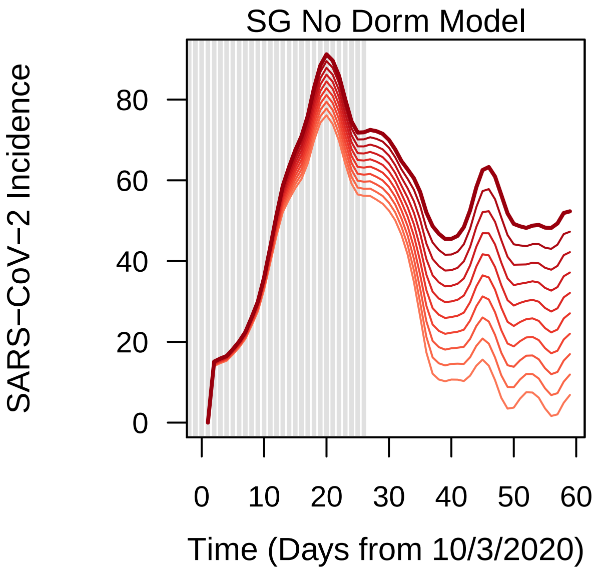

Regression-augmented TVRN for Singapore without COVID-19 Dormitory Case counts

9 Sensitivity Analysis on Serial Interval

Analysis summary

We conducted sensitivity analysis on serial interval estimates by specifying different serial intervals taken from literature while keeping the incubation period fixed at Natalie M Linton et al. 2020's estimate. State coefficient estimates with serial intervals varied are mostly negative, with the direction and effect sizes following that of the primary analysis given in the main manuscript.

9.1 Serial Interval Estimates

Serial Interval (days)	Reference
4.6 (SD=2)	Ferguson et al. 2020
3.96 (95% CI 3.53, 4.39)	Du et al. 2020
4.0 (95% CrI: 3.1, 4.9)	Nishiura, Natalie M. Linton, and Akhmetzhanov 2020
4.6 (95% CrI: 3.5, 5.9)	Nishiura, Natalie M. Linton, and Akhmetzhanov 2020
5.21 (95% CrI: -3.35, 13.94)	Ganyani et al. 2020
3.95 (95% CrI: -4.47, 12.51)	Ganyani et al. 2020

Serial interval estimates taken from literature

9.2 State Coefficient Estimates

	Du[1]		Nishiura[2]		Gayani SG[3]		Gayani CH[4]	
	Mean	95% CrI	Mean	95% CrI	Mean	95% CrI	Mean	95% CrI
IN	-0.124	(-0.151, -0.097)	-0.033	(-0.049, -0.018)	-0.131	(-0.157, -0.105)	-0.119	(-0.143, -0.094)
KH	-0.112	(-0.172, -0.05)	-0.12	(-0.152, -0.081)	-0.102	(-0.162, -0.042)	-0.167	(-0.225, -0.107)
LA	0.046	(0.008, 0.082)	-0.065	(-0.097, -0.035)	0.059	(0.025, 0.095)	0.032	(-0.002, 0.073)
MS	0.017	(-0.002, 0.036)	-0.021	(-0.027, -0.014)	0.021	(0.003, 0.04)	-0.052	(-0.064, -0.039)
MY	0.023	(0, 0.045)	0.018	(-0.007, 0.034)	0.031	(0.007, 0.057)	0.025	(0.001, 0.045)
PH	-0.019	(-0.038, 0.002)	-0.018	(-0.025, -0.01)	-0.016	(-0.035, 0.003)	-0.027	(-0.046, -0.007)
SG	-0.026	(-0.033, -0.019)	-0.003	(-0.008, 0.003)	-0.026	(-0.033, -0.02)	-0.021	(-0.027, -0.014)
TH	-0.118	(-0.143, -0.094)	-0.067	(-0.087, -0.049)	-0.123	(-0.147, -0.099)	-0.12	(-0.143, -0.098)
VN	-0.018	(-0.038, 0.002)	-0.041	(-0.06, -0.022)	-0.012	(-0.032, 0.006)	-0.04	(-0.059, -0.02)

Posterior means and 95% credible intervals for the state coefficient governing change from baseline mobility in residential areas using different serial interval estimates. Incubation period is kept fixed using Linton et Al's estimate.

9.3 Model Fit

	Du[1]		Nishiura[2]		Gayani SG[3]		Gayani CH[4]	
	MAE	R^2	MAE	R^2	MAE	R^2	MAE	R^2
IN	0.32	0.53	0.09	0.80	0.34	0.53	0.27	0.64
KH	0.19	0.47	0.04	0.98	0.20	0.40	0.17	0.76
LA	0.06	0.89	0.02	0.99	0.07	0.91	0.06	0.84
MS	0.44	0.01	0.14	0.72	0.46	0.02	0.41	0.28
MY	0.10	0.61	0.06	0.68	0.11	0.68	0.10	0.63
PH	0.38	0.03	0.16	0.55	0.42	0.02	0.36	0.08
SG	0.28	0.51	0.14	0.05	0.31	0.48	0.27	0.44
TH	0.38	0.45	0.09	0.90	0.42	0.42	0.35	0.57
VN	0.26	0.10	0.05	0.91	0.27	0.04	0.23	0.39

Model fit for the state equation using different serial interval estimates. Incubation period is kept fixed using Linton et Al's estimate.

10 Sensitivity Analysis on Incubation Period

Analysis summary

We conducted sensitivity analysis on incubation period estimates by specifying different incubation period taken from literature while keeping the serial interval fixed at Ferguson et al. 2020's estimate. State coefficient estimates with the incubation period varied are mostly negative, with the direction and effect sizes following that of the primary analysis given in the main manuscript.

10.1 Incubation Period Estimates

Incubation Period (days)	Reference
5.6 (95% CI: 5.0, 6.3)	Natalie M Linton et al. 2020
5.1 (95% CI: 4.5, 5.8)	Lauer et al. 2020
6.4 (95% CrI: 5.6, 7.7)	Backer, Klinkenberg, and Wallinga 2020
4.9 (95% CI: 4.4, 5.5)	Jiang, Rayner, and Luo 2020
5.4 (95% CI: 4.8, 6.0)	Yang et al. 2020

Incubation period estimates taken from literature

10.2 State Coefficient Estimates

	Lauer[1]		Backer[2]		Jiang[3]		Yang[4]	
	Mean	95% CrI	Mean	95% CrI	Mean	95% CrI	Mean	95% CrI
IN	-0.101	(-0.114, -0.087)	-0.106	(-0.122, -0.091)	-0.101	(-0.115, -0.087)	-0.101	(-0.115, -0.086)
KH	-0.163	(-0.201, -0.123)	-0.144	(-0.181, -0.102)	-0.143	(-0.18, -0.112)	-0.15	(-0.193, -0.118)
LA	-0.039	(-0.065, -0.014)	-0.035	(-0.085, 0.011)	-0.031	(-0.059, -0.008)	-0.012	(-0.035, 0.012)
MS	-0.049	(-0.055, -0.042)	-0.055	(-0.062, -0.049)	-0.048	(-0.054, -0.041)	-0.047	(-0.053, -0.041)
MY	0.037	(0.021, 0.055)	0.033	(0.016, 0.056)	0.034	(0.019, 0.05)	0.039	(0.012, 0.054)
PH	-0.038	(-0.046, -0.031)	-0.039	(-0.048, -0.03)	-0.037	(-0.045, -0.03)	-0.037	(-0.045, -0.028)
SG	-0.007	(-0.012, -0.002)	-0.008	(-0.013, -0.003)	-0.007	(-0.012, -0.002)	-0.007	(-0.012, -0.003)
TH	-0.134	(-0.148, -0.12)	-0.138	(-0.155, -0.121)	-0.134	(-0.149, -0.118)	-0.138	(-0.154, -0.124)
VN	-0.062	(-0.081, -0.043)	-0.065	(-0.085, -0.047)	-0.06	(-0.077, -0.042)	-0.062	(-0.082, -0.044)

Posterior means and 95% credible intervals for the state coefficient governing change from baseline mobility in residential areas using different incubation period estimates. Serial interval is kept fixed using Ferguson et Al's estimate.

10.3 Model Fit

	Lauer[1]		Backer[2]		Jiang[3]		Yang[4]	
	MAE	R^2	MAE	R^2	MAE	R^2	MAE	R^2
IN	0.17	0.90	0.19	0.88	0.17	0.90	0.17	0.90
KH	0.12	0.93	0.12	0.90	0.11	0.93	0.10	0.94
LA	0.04	0.96	0.03	0.95	0.03	0.95	0.03	0.72
MS	0.25	0.82	0.26	0.83	0.24	0.82	0.23	0.83
PH	0.26	0.66	0.29	0.58	0.26	0.66	0.26	0.64
SG	0.24	0.11	0.25	0.13	0.23	0.12	0.23	0.13
TH	0.24	0.86	0.27	0.84	0.25	0.86	0.22	0.89
VN	0.13	0.83	0.12	0.84	0.13	0.81	0.12	0.84

Model fit for the state equation using different incubation period estimates. Serial interval is kept fixed using Ferguson et Al's estimate.

References

Backer, Jantien A, Don Klinkenberg, and Jacco Wallinga (2020). "Incubation period of 2019 novel coronavirus (2019-nCoV) infections among travellers from Wuhan, China, 20–28 January 2020". In: *Eurosurveillance* 25.5. DOI: 10.2807/1560-7917.es.2020.25.5.2000062.

Du, Zhanwei et al. (2020). "Serial Interval of COVID-19 among Publicly Reported Confirmed Cases". In: *Emerging Infectious Diseases* 26.6, pp. 1341–1343. DOI: 10.3201/eid2606.200357.

Ferguson, Neil et al. (2020). "Report 9: Impact of non-pharmaceutical interventions (NPIs) to reduce COVID19 mortality and healthcare demand". In: *MRC Centre for Global Infectious Disease Analysis, COVID-19 Reports*. DOI: 10.25561/77482.

Ganyani, Tapiwa et al. (2020). "Estimating the generation interval for coronavirus disease (COVID-19) based on symptom onset data, March 2020". In: *Eurosurveillance* 25.17. DOI: 10.2807/1560-7917.es.2020.25.17.2000257.

Jiang, Xuan, Simon Rayner, and Min-Hua Luo (2020). "Does SARS-CoV-2 has a longer incubation period than SARS and MERS?" In: *Journal of Medical Virology* 92.5, pp. 476–478. DOI: 10.1002/jmv.25708.

Lauer, Stephen A. et al. (2020). "The Incubation Period of Coronavirus Disease 2019 (COVID-19) From Publicly Reported Confirmed Cases: Estimation and Application". In: *Annals of Internal Medicine* 172.9, pp. 577–582. DOI: 10.7326/m20-0504.

Linton, Natalie M et al. (2020). "Incubation period and other epidemiological characteristics of 2019 novel coronavirus infections with right truncation: a statistical analysis of publicly available case data". In: *Journal of Clinical Medicine* 9.2, p. 538. DOI: 10.3390/jcm9020538.

Nishiura, Hiroshi, Natalie M. Linton, and Andrei R. Akhmetzhanov (2020). "Serial interval of novel coronavirus (COVID-19) infections". In: *International Journal of Infectious Diseases* 93, pp. 284–286. DOI: 10.1016/j.ijid.2020.02.060.

Yang, Lin et al. (2020). "Estimation of incubation period and serial interval of COVID-19: analysis of 178 cases and 131 transmission chains in Hubei province, China". In: *Epidemiology and Infection* 148. DOI: 10.1017/s0950268820001338.
