# Supplementary material for: Revealing regional disparities in the transmission potential of SARS-CoV-2 from interventions in Southeast Asia
Source: Proc Biol Sci. 2020 Aug 26;287(1933):20201173. doi: 10.1098/rspb.2020.1173 (PMC7482285; doi:10.1098/rspb.2020.1173)
Supplement: Appendix 2 [file rspb20201173supp2.docx]

| **Country** | **Social Distancing Measures** | **Implementation Date**  **(as of 18-05-2020)** |
| --- | --- | --- |
| **Malaysia** | *Movement Control Order* | |
|  | - General prohibition of mass movements and gatherings across the country including religious, sports, social and cultural activities. | 18-03-2020 to 04-05-2020  Extended to 12-05-2020 on 23-04-2020 |
|  | - Closure of borders | 18-03-2020 to 04-05-2020  Extended to 14-04-2020 on 25-03-2020 |
|  | - Closure of all schools and institutions | Extended to 28-04-2020 on 10-04-2020 |
|  | - Closure of all government and private premises except those involved in essential services | Extended to 12-05-2020 on 23-04-2020 |
|  | *Enhanced Movement Control Order* |  |
|  | - All residents and visitors within the area are forbidden from exiting their home - Lockdown on specific area - All businesses are shut down; adequate food supplies is given to residents by authorities and medical base is established within the area | 27-03-2020 onwards  (14-day restriction on specific areas if large cluster is detected*)* |
|  | *Conditional Movement Control Order (Relaxed Lockdown)* | |
|  | - All public transport services resume - Two family members will be allowed to buy food and other daily essentials - Reopening of certain businesses | 04-05-2020 to 12-06-2020 |
| **Laos** | - Closing all schools and many shops - Closing all border checkpoints, and suspending the issuance of tourist visas for at least 30 days | 17-03-2020 to 29-03-2020 |
|  | - Residential lockdown imposed with limited exceptions - Government and Civil Service employees to stop work until April 11th - Prohibition of gatherings of more than 10 people - Border closures except for transportation of Goods | 30-03-2020 to 19-04-2020  Extended to 03-05-2020 on 16-04-2020 |
|  | *Relaxation of Measures (Temporary Basis)*   - If additional COVID-19 cases are reported in one province, that province will return to a lockdown state and will be placed under the authority of the provincial taskforce. If infections are reported in two or more provinces, Laos will return to a full national lockdown, and the country's [original lockdown order](https://www.tilleke.com/resources/laos-imposes-sweeping-covid-19-lockdown-measures) will be reinstated. - People are now authorised to leave their homes and travel within provinces - Working remotely is recommended, for workplaces that resume normal operations, prevention measures must be in place | 04-05-2020 to 17-05-2020 |
| **Cambodia** | - Closure of all Schools and cancellation of Songkran | 16-03-2020 |
|  | - Closure of Entertainment Establishments and Religious Places | 18-03-2020 |
|  | - Border Closure | 23-03-2020 |
|  | - Lockdown of all district and provincial Borders | 09-04-2020 to 16-04-2020 |
|  | - Relaxing of Lockdown Measures | 16-04-2020 Onwards |
| **Singapore** | - Closure of certain entertainment venues - Groups must not exceed 10 people - At least 1m physical spacing in public venues (e.g. queuing, eating) - Tuition and enrichment centres, faith-based activities, events are suspended - SHNs for UK/US returnees | 27-03-2020 to 06-04-2020 |
|  | Circuit Breaker Measures   - Only takeaway and delivery are is allowed at all food places - Physical retail outlets offering non-essential services are closed - Closure of schools - Closure of workplaces unless deemed essential services - Closure of all recreational venues and places of worship - Government advises public to wear masks - Public is advised to stay home unless for essential purposes | 07-04-2020 to 21-04-2020 |
|  | Stricter/Extended Circuit Breaker   - Public advised to go out alone - More businesses are closed (e.g. stalls selling mostly beverages/desserts, barbers, hairdressers, TCM/acupuncture) - Additional restrictions placed on businesses (e.g. optician visits by appointment only) - Controlled access at areas susceptible to crowding (e.g markets) - Increased enforcement at work premises | 22-04-2020 to 11-05-2020 |
|  | Extended Circuit Breaker   - Opening of selected retail businesses - Home-based food businesses are allowed to operate | 12-05-2020 to 01-06-2020 |
| **Indonesia** | Large Scale Social Restrictions   - Include the closure of schools and workplaces, restrictions on religious activities and restrictions on activities in public places. - The National Police could take legal steps against anyone violating the rules under the large-scale social-distancing measures.   Regional Quarantine   - Border restrictions in specific areas. Under the public health emergency measures, the central government allows regional administrations to make a request to close their borders, but not large-scale areas.   Public Health Emergency Declared | 15-03-2020 to 23-04-2020 |
|  | Lockdown announced   - The Transport Ministry suspended all domestic flights and ferry operations, with exceptions for commercial cargo, medical and migrant worker evacuation, state officials, diplomats and residents of small islands. - All public and private vehicles, including cars, motorcycles and buses, are not permitted to travel outside the Greater Jakarta area for the next five weeks, with dozens of checkpoints established on main toll roads and other access and egress points to enforce the policy and to restrict movement within the metropolis itself. - Stricter Social Distancing Measures implemented | 24-04-2020 to 01-06-2020 |
| **Thailand** | Emergency Decree Invoked   - Closures of various businesses and venues in most parts of the country, a mask requirement on public transportation, and travel restrictions to and from some provinces. - Inclusion of night time curfew of between 10pm and 4am and inbound travel restrictions | 26-03-2020 to 30-04-2020 |
|  | Extension of State of Emergency   - The night time curfew of between 10pm and 4am and inbound travel restrictions will remain in place. - An extension to the ban on all inbound commercial flights except for repatriation, cargo and emergency landings | Extended to 31-05-2020 on 28-04-2020 |
|  | Easing of Lockdown Measures   - Restaurants, hair salons, parks, markets, open-air sports venues such as tennis courts, golf courses, shooting and archery ranges, and pet grooming salons and nurseries are reopening nationwide - Social distancing rules are still in effect and are strictly adhered to. Restaurants need to seat their customers 1.5m apart from one another - At salons and barbershops, waiting inside is not allowed. Each customer must make an appointment, while shops are required to be cleaned for 20 minutes after each one of the two-hour slots. | From 03-05-2020 Onwards |
| **Myanmar** | Lockdown Announced   - All incoming travelers from Thailand and Malaysia through borderline gates are required to be home quarantined for 14 days. - All government and private schools, universities and entertainment centers are closed. Supermarkets and shopping centers are still opened. Restaurants are only for delivery service. - Some private offices are closed until the end of April, while most switch to working from home or partially. - Public transport is still operating but at lower capacity. The express Bus Transportation reduce the number of passengers to half - All the airports are locked down from March 30 to May 15, 2020. | 23-03-2020 to 15-05-2020 |
|  | Lockdown Extension   - The government has placed 10 townships in Yangon under semi-lockdown. - The authorities have announced the 10pm-4am curfew imposed in all 45 townships, will remain effective beyond May 15 until June 18, according to the Yangon regional government on April 23, 2020 | Extended to 18-06-2020 on 23-04-2020 |
| **Vietnam** | - Closure of borders - Large-scale events are cancelled - Public transport passengers are required to fill out health declaration forms | 20-03-2020 onwards |
|  | - Closure of non-essential businesses such as entertainment venues - Prohibition of gatherings involving more than 10 | 25-03-2020 to 31-03-2020 |
|  | - Stricter measures implemented - self-isolation and restricting people from leaving homes except for food and medicines. - The gathering of more than two people is also banned while also keeping a distance of two meters when outside. | 01-04-2020 to 22-04-2020 |
|  | - Gradual relaxation of tightened measures | 23-04-2020 to 03-05-2020 |
|  | - Gradual reopening of schools - Resumption of non-essential businesses except for clubs and karaoke parlours with preventive measures - Resumption of religious activities with preventive measures for large events | 04-05-2020 onwards |
| **The Philippines** | - Closure of schools - Prohibition of mass gatherings | 09-03-2020 onwards |
|  | Enhanced Community Quarantine   - Home quarantine measures implemented - public is only to leave the home for essential needs - Non-essential work and transport is halted | 16-03-2020 to 30-04-2020 |
|  | General Community Quarantine   - Measures are eased in certain low-risk areas - Public transportation is allowed at a reduced capacity and select business are to reduce capacity - Only select stalls and stores in shopping malls are allowed to open | 01-05-2020 onwards |
